# The perioperative period of liver transplantation from unconventional extended criteria donors: data from two high-volume centres

**DOI:** 10.1186/s12871-022-01932-x

**Published:** 2022-12-15

**Authors:** Claudia Pescarissi, Beatrice Penzo, Davide Ghinolfi, Quirino Lai, Lucia Bindi, Riccardo DeCarlis, Fabio Melandro, Emanuele Balzano, Paolo DeSimone, Luciano DeCarlis, Andrea DeGasperi, Italian Collaborative Group on Liver Transplantation from Extended Criteria Donation, Ombretta Amici, Gabriella Amorese, Caterina Barbaglio, Jacopo Belfiore, Massimo Bisà, Andrea Brunetti, Daniela Camera, Niccolò Castellani Nicolini, Gabriele Catalano, Eva Cibelli, Stefania Colombo, Giovanni Consani, Simone DiMatteo, Massimo Esposito, Elena Guffanti, Ernestina Mazza, Tommaso Mazzanti, Luca Meacci, Pietro Molinari, Laura Petrò, Giorgia Pratesi, Manlio Prosperi, Francesca Puccini, Elena Roselli, Ilenia Scaffidi, Alicia Spelta, Riccardo Taddei, Giovanni Tincani, Francesco Torri, Giandomenico L. Biancofiore

**Affiliations:** 1 Transplant Anesthesia and Critical Care, University of Pisa Medical School Hospital, Via Paradisa 2 – 56124, Pisa, Italy; 2 Department of Anesthesia, ASST GOM Niguarda, Milan, Italy; 3 Hepatobiliary Surgery and Liver Transplantation, University of Pisa Medical School Hospital, Pisa, Italy; 4 Sapienza University of Rome, AOU Umberto I Policlinico of Rome, Rome, Italy; 5 Department of Surgery, ASST GOM Niguarda, Milano, Italy; 6 School of Medicine, University of Milano-Bicocca, Milano, Italy

**Keywords:** Liver transplantation, Organ donation, Perioperative care, Complications, Intensive care unit

## Abstract

**Background:**

As literature largely focuses on long-term outcomes, this study aimed at elucidating the perioperative outcomes of liver transplant patients receiving a graft from two groups of unconventional expanded criteria donors: brain dead aged > 80 years and cardiac dead.

**Methods:**

Data of 247 cirrhotic patients transplanted at two high volume liver transplant centers were analysed. Confounders were balanced using a stabilized inverse probability therapy weighting and a propensity score for each patient on the original population was generated. The score was created using a multivariate logistic regression model considering a Comprehensive Complication Index ≥ 42 (no versus yes) as the dependent variable and 11 possible clinically relevant confounders as covariate.

**Results:**

Forty-four patients received the graft from a cardiac-dead donor and 203 from a brain-dead donor aged > 80 years. Intraoperatively, cardiac-dead donors liver transplant cases required more fresh frozen plasma units (*P* < 0.0001) with similar reduced need of fibrinogen to old brain-dead donors cases. The incidence of reperfusion syndrome was similar (*P* = 0.80). In the Intensive Care Unit, both the groups presented a comparable low need for blood transfusions, renal replacement therapy and inotropes. Cardiac-dead donors liver transplantations required more time to tracheal extubation (*P* < 0.0001) and scored higher Comprehensive Complication Index (*P* < 0.0001) however the incidence of a severe complication status (Comprehensive Complication Index  ≥ 42) was similar (*P* = 0.52). ICU stay (*P* = 0.97), total hospital stay (*P* = 0.57), in hospital (*P* = 1.00) and 6 months (*P* = 1.00) death were similar.

**Conclusion:**

Selected octogenarian and cardiac-dead donors can be used safely for liver transplantation.

## Background

Despite significant improvements, the transplant community still complains of an increasing disparity between cadaveric donors’ availability and actual recipients demand [1]. Hence, there is a growing need to identify new organ sources [2, 3].

Expanded criteria donors (ECDs) represent a spectrum of donors which has been traditionally considered as “suboptimal” or even “marginal” because of potential poor outcomes after liver transplantation (LT) [2, 3]. In particular, elderly donor livers, generally defined as > 70 years, are still quite frequently discarded for fear of morphological and functional alterations linked to aging [3, 4]. However, recent studies challenge this dogma and show that with increasing experience and careful selection of both donor and recipient, even eighty-year-old brain-dead donor (DBD) grafts can become a safe and efficient option to increase the donor pool [1–5].

Donors after cardiac death (DCDs) are considered to be expanded-criteria because they are exposed to a variable period of warm ischemia before organ preservation. This leads to an increased risk of ischemia-reperfusion injury (IRI) and peri-LT complications [6, 7]. Nevertheless, also due to the expanding use of machine perfusion (MP) techniques, interest in DCD-LT has grown in recent years thanks to their encouraging results and the number of institutions offering this procedure is increasing [6–8]. However, although expanding, the use of octogenarian and DCD liver grafts for transplantation is not yet common practice at transplant centers and, also according to a large consensus, specific experience and skills are recognized as important factors for best results [1–8].

In summary, in the effort to increase the donor pool, uncommon types of LT donors have been increasingly considered in recent years with a growing number of LT procedures using liver grafts from DCDs and very old DBDs [3, 4]. Interestingly, the literature focuses primarily on their long-term outcomes and only limited data are available about the perioperative period which, however, is increasingly recognized as relevant [1, 5, 9, 10]. Therefore, after a preliminary balancing, we herein report and compare the operating theatre and Intensive Care Unit (ICU) courses of patients who received a LT from a DCD or a DBD aged ≥ 80 years.

## Methods

This was an observational, retrospective cohort study investigating the clinical data of patients undergoing LT at two high volume Italian national Centres. The study was approved by the ethic committee and the Strengthening the Reporting of Observational Studies in Epidemiology (STROBE) guidelines were followed to create the study. All patients receiving a LT from DCDs and old (≥ 80 years) DBDs were included and stratified in two groups. The main outcome of the study was the presence of one of more severe complications determining a Comprehensive Complication Index (CCI) ≥ 42 during the ICU stay. Secondary outcomes were a) surrogates of early graft function; b) need for transfusions; c) need for inotropes; and d) extubating timing, all of these evaluated during the ICU stay.

### DCDs management and eligibility

As per Italian legislation, DCD donation after cardiac death requires 20 min of continuous, flat-line electrocardiogram to proceed to declaration of death for both controlled (cDCD) and uncontrolled (uDCD) donors [11]. Veno-arterial ECMO (ECMO-machine, Maquet, Rastatoth, Germany) for abdominal normothermic regional perfusion (NRP) after cardiac death declaration was used in all donors as already described [12].

Potential DCDs were considered eligible for procurement according to the following shared criteria: 1) no absolute contraindication according to the Italian National Transplant Centre guidelines [11]; 2) age ≤ 70 years for uDCD, no age limit in cDCD; 3) witnessed cardiac arrest; 4) NRP flow > 2.0 L/min; 5) preliminary acceptable liver graft appearance at surgical inspection at the time of harvesting and 5) at least, two of the following: a) total warm ischemia time ≤ 170 min;b) alanine aminotransferase (ALT) < 1000 UI/L;c) stable or decreasing lactate concentration during NRP. Liver biopsy at procurement was mandatory and grafts were discarded if any of the following was present: macro-vescicular steatosis > 30%; fibrosis > 2 as per Ishak’s score [13]; necrosis > 10%, and severe macroangiopathy (as per arteriolar thickening > 60%) [12].

### Machine perfusion techniques

Grafts were stored at 4 °C, shipped and then re-perfused ex-vivo, with a hypo- or a normo-thermic technique (LiverAssist®, OrganAssist®, Groeningen;Netherlands). When NMP was adopted, grafts were considered eligible for transplant based on: 1) a perfusate lactate clearance irrespective of its baseline value, 2) their gross macroscopic appearance with uniform vascularization and stable flow.

### DBDs

Eligibility to liver donation was evaluated as per our institutional policy and according to the Italian National Transplant Agency guidelines [11]. Reasons for discarding a DBD liver graft were donor HIV-positivity,history of melanoma or lymphoproliferative disease,or any intractable systemic infection. History of malignancy within 5 years (10 years for breast cancer), HCV-positivity and hepatitis B surface antigen (HBsAg)-positivity required donor–recipient matching and evaluation of urgency and benefit of transplantation [11]. Liver graft biopsy was performed on demand based on surgical evaluation at procurement. All grafts were routinely evaluated on the back table before LT for vessel patency and anatomical variants.

### Recipients

Based on surgeon’s preference, LTs were performed using conventional or piggy-back techniques. Standard anesthetic management for LT at the two institutions was used. Briefly, anesthesia was induced and maintained with intravenous and inhalational anesthetics combined with IV fentanyl or remifentanil and muscle relaxants. Hemodynamic monitoring included invasive arterial blood pressure measurement and the use of a pulmonary artery catheter with semi-continuous Cardiac Output measurement capacity (Edwards Life Sciences LLC, Irvine, CA, USA). Coagulation was managed under thromboelastographic guidance as already described [14]. After surgery, patients were transferred to the Intensive Care Units (ICUs) of the 2 centers where anesthesiologists are in charge.

### Data, measurements and definitions

Data were retrospectively extracted from prospectively maintained databases at the participating centers. All data were pseudonymized.

Intraoperative measurements included: duration of warm and cold ischemia, number of blood transfusions and of patients needing hemodynamic pharmacological support. Postoperative variables included: amounts of blood transfusions, number of patients where a fast track recovery with early tracheal extubation was possible, length of postoperative mechanical ventilation, length of ICU and hospital stay, number of post-LT re-interventions, quality of graft recovery, patients survival at hospital discharge and 6 months afterwards. The last follow-up date was 30 September 2021. Postoperative complications while in the ICU, defined as any deviation from the expected postoperative course [15], were individually assessed. Severity of complications was graded by the Clavien-Dindo (CD) classification [15, 16]. To determine the seriousness of the postoperative course, at patients’ ICU discharge, the Comprehensive Complication Index (CCI) [17] was calculated by an online calculator (www.assessurgery.com/about_CCI-calculator/). The CCI grades complications by the CD classification and implements every occurred weighted complication after an intervention summarizing postoperative morbidity on a numerical scale from 0 (no complication) to 100 (death). We ranked multiorgan as CD grade IVb, primary non-function (PNF), identified as a liver failure observed for non-technical reasons within seven days after surgery as CD grade IVa [18]. Cold ischemia time (CIT) was classically defined as the period from the donor aortic clamping to the liver out of ice. Warm ischemia time (WIT) was calculated using the time frame from the liver out of ice to the time of arterial reperfusion. Reperfusion syndrome (RS) was defined as a mean arterial blood pressure 30% lower than the previous value immediately at the end of the anhepatic stage lasting for at least 1 min within 5 min after unclamping [19]. Early graft function was defined according to Olthoff’s criteria as bilirubin ≥ 10 mg/dL on day 7, international normalized ratio ≥ 1.6 on day 7 and alanine or aspartate aminotransferases > 2000 IU/L within the first 7 days [20]. Acute kidney injury (AKI) was defined by the AKIN criteria [21].

### Statistical analysis

Continuous variables were reported as medians and 25th -75th percentile. Categorical variables were described as numbers and percentages. Comparisons between groups were made using Fisher’s exact test or chi-square test for categorical variables, as appropriate. Mann-Whitney was used for continuous variables. Missing data relative to study covariates always involved less than 10% of patients. In all the cases, missing data were handled with a single imputation method. In detail, a median of nearby points imputation was adopted. The median instead of the mean was adopted due to the skewed distribution of the managed variables. The entire population was divided in two groups according to the donor characteristics (DCD and DBD aged > 80 years). With the intent to compensate for the non-randomized design of this retrospective study, the two groups were “balanced” using a stabilized inverse probability therapy weighting (IPTW). A propensity score for each patient on the original population was generated. The score was created using a multivariate logistic regression model considering a CCI ≥ 42 (no versus yes) as the dependent variable. We selected eleven possible clinically relevant confounders as covariates: patient male sex, patient age, patient BMI, viral liver disease, MELD, patient count of platelets, donor BMI, donor ALT peak, donor bilirubin peak, WIT, CIT. All the covariates were available before the end of the LT procedure to avoid the risk of a possible immortal time bias in covariate selection. With the intent to reduce the artificial modification of the sample size in the pseudo data, we used stabilized weights (SW) according to the formula: SW = p /PS for the study group, and SW = (1-p)/(1-PS) for the control group where p is the probability of etiology without considering covariates and PS is the propensity score. Because p-values can be biased from population size, results from the comparisons between covariates subgroups were reported as effect size (Cohen’s D value): values lower than |0.1| indicated very small differences between means, values between |0.1| and |0.3| indicated small differences, values between |0.3| and |0.5| indicated moderate differences, and values greater than |0.5| indicated considerable differences. Variables with a *p* < 0.05 were considered statistically significant. Statistical analyses and plots were run using the SPSS statistical package version 27.0 (SPSS Inc., Chicago, IL, USA).

## Results

Between 1 and 2015 and 30 March 2020, 247 LT patients merging the inclusion criteria of the present study were transplanted at the 2 centers. Forty-four recipients (DCD-LT Group) received a graft from a cardiac-dead donor (39 uncontrolled and 5 controlled) and 203 (old DBD-LT Group) from a brain-dead donor aged ≥ 80 year. After a median post-LT hospital length of stay of 15 days (25th -75th percentile = 12–20), 235 (97.5%) patients were discharged: of them, 6 (2.5%), and 23 (10.0%) patients died at 6 and 12 months after LT, respectively. Eight (3.3%) patients were retransplanted during the first year after LT. The characteristics of the investigated population were reported in the Table 1. As expected, the DCD Group was substantially different with respect to the compared old DBD Group. As an example, the patient in the DCD Group was older, with a higher BMI, and with a tumoral underlying liver disease as the main indication to transplantation. As for the donors in the DCD Group, they had a predictable shorter ICU stay, and higher transaminases peaks respect to the old DBD Group. The totality of the DCD patients had donors experiencing cardiac arrest episodes, a need for biopsy for evaluating the graft quality, and the use of a perfusion machine before graft implantation. The median WIT was longer, and the median CIT was shorter in the DCD Group.

**Table 1:** Characteristics of the investigated population

**Variables**	**DCD-LT** **(*n* = 44)**	**Old DBD-LT** **(*n* = 203)**	***P*-value**
	**Median (**25th -75th percentile**) or n (%)**		
**LT Reciepint**			
** Male sex**	39 (88.6)	156 (76.8)	0.20
** Age, years**	58 (54–64)	56 (52–61)	0.02
** BMI**	27 (24–29)	24 (23–27)	0.003
** HCC, n**	28 (63.6)	82 (40.3)	0.01
** Viral liver disease, n**	9 (20.5)	70 (34.4)	0.08
** MELD**	12 (10–17)	12 (9–16)	0.19
** Platelets x 1,000/ µL**	87.5 (60.3-137.5)	77.0 (58.5–120.0)	0.23
**Donor**			
** Male sex**	41 (93.2)	78 (38.4)	< 0.0001
** Age, years**	56 (47–61)	83 (81–85)	< 0.0001
** BMI**	26 (25–28)	25 (23–27)	0.001
** ICU stay, days**	1 (0–3)	3 (1–5)	< 0.0001
** sAST peak (IU/mL** ^**− 1**^ **)**	235 (62–481)	33 (25–51)	< 0.0001
** sALT peak (IU/mL** ^**− 1**^ **)**	41 (27–157)	23 (15–34)	< 0.0001
** sBilirubin peak (mg/ dl** ^**− 1**^ **)**	0.6 (0.3-1.0)	0.8 (0.6–1.1)	0.027
** Cardiac arrest episodes, n**	44 (100.0)	6 (3.0)	< 0.0001
** Patients on inotropes, n**	39 (88.6)	161 (79.3)	0.38
** Biopsy, n**	44 (100.0)	136 (67.0)	< 0.0001
** Use of perfusion machines, n**	44 (100.0)	0 (-)	< 0.0001
**Transplantation**			
** WIT, minutes**	92 (69–141)	78 (68–90)	0.02
** CIT, minutes**	348 (271–418)	440 (391–500)	< 0.0001

*DCD* deceased cardiac donor, *DBD* deceased brain donor, *I LT* liver transplantation, *QR* interquartile ranges, *n* number, *BMI* body mass index, *HCC* hepatocellular cancer, *HCV, MELD* model for end-stage liver disease, *ICU* intensive care unit, *sAST* serum aspartate aminotransferase, *sALT* serum alanine aminotransferase, *WIT* warm ischemia time, *CIT* cold ischemia time

### Stabilized IPTW effect

With the intent to minimize the effect of selection biases caused by the non-randomized design of this retrospective study, the DCD-LT Group was artificially balanced with the old DBD-LT Group using a stabilized IPTW method. As reported in Table 2, the DCD-LT Group was efficaciously “balanced” for the 11 potential confounders adopted. In detail, when the DCD-LT Group was compared with the old DBD-LT Group before the IPTW balancing, no variable showed very small differences, five small, two moderate, and four considerable differences. After the IPTW, two variables showed very small differences, four small, four moderate, and only one a considerable difference (i.e., CIT), although this latter difference passed from more than one SD difference to a value inferior to one SD (from 1.05 to 0.66). Thanks to the stabilized IPTW, no relevant modification of the initial sample size was observed in the pseudo population, with the DCD-LT Group passing from 44 to 43 cases, and the old DBD Group passing from 203 to 197 cases.

**Table 2:** Effect of stabilized IPTW in the population on the variables used for balancing the DCD-LT with the Old DBD-LT Group

**Variables**	**Pre-IPTW** **Cohen’s D-value**	**Post-IPTW** **Cohen’s D-value**
Patient male sex	-0.32	-0.28
Patient age	0.29	0.26
Patient BMI	0.43	-0.31
Viral liver disease	-0.58	-0.36
MELD	0.18	-0.01
Patient count of platelets	0.26	0.08
Donor BMI	-0.81	0.47
Donor sAST peak	0.78	0.27
Donor bilirubin peak	-0.19	-0.10
WIT	0.24	0.44
CIT	-1.05	0.66

*IPTW* inverse probability therapy weighting, *DCD* deceased cardiac donor, *DBD* deceased brain donor, *LT* liver transplantation, *n* number, *SD* standard deviation, *BMI* body mass index, *MELD* model for end-stage liver disease, *sAST* serum aspartate aminotransferase, *WIT* warm ischemia time, *CIT* cold ischemia time

### Comparison between DCD and old DBD Group

The results of the perioperative clinical course observed in the DCD-LT and Old DBD-LT Group after their IPTW balancing are reported in Table 3. During LT surgery, DCD-LT cases required more FFP (6 vs. 4 units; *P* < 0.0001) with similar reduced need of fibrinogen to old DBD-LTs. The incidence of RS was also similar (11.6% in the DCD group Vs 14.3% in the old DBD group, *P* = 0.80). In the ICU, both the study groups presented a comparable and low need for blood transfusions, renal replacement therapy and inotropes. DCD-LTs showed higher post-LT AST peak (5343 Vs 768, *p* < 0.0001), required more time to tracheal extubation (*P* < 0.0001) and scored higher CCI (8.7 vs. 0.0; *P* < 0.0001). Quite surprisingly, the number of patients with a severe complication status (CCI ≥ 42) was similar in both groups (14.0 in DCD-Lys vs. 18.7% in old DBD_LTs; *P* = 0.52). ICU stay (median = 3 days; *P* = 0.97) and total hospital stay (median 15 vs. 16 days; Pe = 0.57) were similar in the two groups. Rates of re-LT (*P* = 0.57), death during the hospital stay (*P* = 1.00), and death at 6 months (*P* = 1.00) were also similar between the two groups. These results may reflect the advanced level of perioperative care acquired by the participating institutions.

**Table 3:** Post-transplant clinical course observed in the DCD-LT and Old DBD-LT Group (pseudo populations after IPTW balancing)

**Variables**	**DCD-LT (*n* = 43)**	**Old DBD-LT (*n* = 197)**	***P*-value**
	**Median (**25th -75th percentile**) or n (%)**		
**Intra-operative**			
** PRBC Units, n**	2 (2–2)	2 (0–4)	0.75
** FFP Units, n**	6 (6–7)	4 (0–5)	< 0.0001
** Fibrinogen Concentrate, grams**	0 (0–0)	0 (0–0)	0.97
** Platelets Units, n**	0 (0–0)	0 (0–0)	0.13
** Reperfusion Syndrome, n**	5 (11.6)	29 (14.7)	0.80
**ICU stay**			
** sAST peak (IU/mL** ^**− 1**^ **)**	5343 (3925–5343)	768 (449–1177)	< 0.0001
** sALT peak (IU/mL** ^**− 1**^ **)**	423 (423–1672)	436 (329–662)	0.89
** sBilirubin peak (mg/ dl** ^**− 1**^ **)**	3.0 (3.0-5.1)	3.8 (2.4–4.8)	0.90
** INR**	1.40 (1.40–2.10)	1.55 (1.46–1.85)	0.04
** ICU stay, days**	3 (3–3)	3 (3–3)	0.97
** PRBC Units, n**	0 (0–0)	0 (0–0)	0.006
** FFP Units, n**	0 (0–0)	0 (0–0)	0.01
** Platelets**	0 (0–0)	0 (0–0)	0.13
** Inotropes**	5 (11.6)	22 (10.8)	0.79
** Time of extubation, days**	1 (1–2)	0 (0–0)	< 0.0001
** Patietns on CRRT, n**	2 (4.7)	9 (4.4)	1.00
** CCI score** ** Patients with CCI score ≥ 42, n**	8.7 (8.7–8.7) 6 (14.0)	0 (0-20.9) 38 (19.2)	< 0.0001 0.52
**Post-ICU clinical course**			
** Hospital stay, days**	15 (15–15)	16 (12–22)	0.57
** Re-LT, n**	2 (-)	6 (3.0)	0.59
** Patient death during hospital stay,n**	1 (-)	3 (1.5)	1.00
** Patient death at 6 months, n**	1 (-)	3 (1.5)	1.00

*DCD* deceased cardiac donor, *DBD* deceased brain donor, *LT* liver transplantation, *IQR* interquartile ranges, *n* number, *PRBC* packed red blood cells, *FFP* fresh frozen plasma, *ICU* intensive care unit, *sAST* serum aspartate aminotransferase, *sALT* serum alanine aminotransferas, *INR* international normalized ratio, *CRRT* continuous renal replacement therapy, *CCI* comprehensive complication index, *LT* liver transplantation

## Discussion

In recent years, the indications for LT have expanded significantly without a concomitant increase in the number of available donors. Some strategies for reducing the mismatch between supply and demand involve policy changes, while others depend more directly on a better knowledge of the opportunities available to increase the donor pool [3, 4]. Therefore, in-depth understanding of all the different phases of the transplant procedure is essential in particular when still not common ECDs, such as octogenarian DBDs and DCDs, are used.

Summing up our results, we found that: a) the intraoperative course was similarly uneventful in the two groups and only fresh frozen plasma transfusions were different; b) the attending anesthesiologists used more caution in considering DCD-LT recipients for a fast track recovery with early tracheal extubation; c) in the ICU, the 2 groups showed comparable, reduced, need for blood transfusions, inotropes and renal replacement therapy; d) DCD-LTs scored higher CCI in the ICU but the severe complication status (CCI ≥ 42) was similar to old DBD-LTs; e) rates of graft dysfunction and mortality were also similarly low. These are interesting findings because they further encourage anesthesiologists and intensivists not to consider LTs using these two particular and uncommon categories of ECDs as necessarily associated with a dramatically severe perioperative course and outcomes.

With regard to donors age, it is well known that the functional impact of aging is less pronounced on the liver than on kidneys or the heart [22]. Thus, the utilization of elderly donors is rising worldwide due to the increasing evidence that acceptance of an old graft confers a survival benefit for waitlist candidates across all MELD scores, particularly in high MELD candidates [22–24]. Donors after cardiac death are considered ECDs because they undergo a period of warm ischemia before organs’ preservation with a higher risk of IRI, graft dysfunction and complications than DBDs [6, 7]. This problem can be minimized by well-organized pathways and techniques of in situ organ preservation coupled to ex situ post-explantation resuscitation and repair which are providing encouraging results in selected recipients compared to DBD-LT [3, 6, 7, 25–29].

We are aware that the perioperative period of LT with ECD organs, in particular octogenarian DBD and DCD donors, can result more resource intensive than for standard DBD organs. Furthermore, they are potentially exposed to higher rates of complications with increased costs [30]. However, we demonstrate equivalent perioperative outcomes and similar complications adding more evidence to the increasingly robust and consistent prior research that suggests that liver allografts from older donors and DCDs provide a survival benefit [30–32].

In order to make our investigation as unbiased as possible, we rated complications according to objective tools like the CD classification and the CCI score. The CD classification was first described in 2004 and nowadays represents the most frequently used grading system for weighting postoperative morbidity [9, 18]. However, although objective, simple, and reproducible, the CD classification is affected by the limitation of scaling the whole postoperative course by the single most severe occurred event [16, 17]. Therefore, to overcome this problem, the CCI has been proposed which gathered a wide agreement about its capability to capture more precisely the overall morbidity burden. In fact, in a multicenter study of a total of more than 1700 LT patients, Lai et al. found the CCI with a very good diagnostic power for 90-day and 1-year graft loss in different sets of patients, indicating better accuracy with respect to other pre- and post-LT scores [18]. Moreover, Staiger et al., in a population of 479 patients undergoing major general surgery including LT, outlined that the CCI can be a useful warning signal for overall high morbidity by 90 days [9]. Finally, Castanedo et al., in a study of 164 LT patients where different score systems were assessed, showed that the CCI, as a measure of overall morbidity, was an independent negative predictive factor of long-term survival [10]. The sum of all of these observations suggests the CCI as a useful tool for objectively alerting caregivers to any worsening of patients’ clinical conditions. In our experience, although the CCI score resulted higher in DCD-LT recipients, its computation failed to predict worse ICU outcomes, namely longer ICU stay and mortality. This can be interpreted with the high level of diagnostic awareness, experience and therapeutic readiness available in the two high volume centers and, in particular, outlines the crucial role of anesthesiologists and intensivists in determining outcomes of LTs using these particular groups of ECDs.

We are aware of some limitations of our study. Our DCD-LT sample size is relatively small. However, it has to be outlined that only approximately 4% of all of the LTs performed in Italy in the last 2 years used DCD grafts [33]. To enable a comparison of outcomes reducing bias due to confounders, we matched patients ensuring an equal distribution among the groups of the variables believed to be confounding. Moreover, we minimized the effect of selection biases caused by the non-randomized design of this retrospective study by artificially balancing the DCD-LT Group with the old DBD-LT Group using a stabilized IPTW method. Finally, although we used an objective tool to rate the severity of complications, some categorizations were arbitrary. Thus, the frequency of specific complications should be interpreted with caution.

## Conclusion

In summary, the transplant community strives to find ways to help to reduce the gap between donor liver grafts availability and patients on the LT waiting list. Our data reinforce the concept that there is a significant potential to increase the supply and utilization of ECDs for transplantation and that the perioperative period of LT from selected octogenarian and DCD donors can be safe. To this end, a careful perioperative management provided by experienced anesthesiologists and intensivists is crucial.

## Data Availability

The datasets used and/or analysed during the current study are available from the corresponding author on reasonable request.

## Abbreviations

CCI: Comprehensive Complication Index
CD classification: Clavien-Dindo classification
DBD: brain-dead donor
DCD: donor after cardiac death
ECD: Expanded criteria donor
LT: liver transplantation
MP: machine perfusion
